# Establishment of a Sensitive and Reliable Droplet Digital PCR Assay for the Detection of *Bursaphelenchus xylophilus*

**DOI:** 10.3390/plants13192701

**Published:** 2024-09-26

**Authors:** Yu Su, Xuedong Zhu, Haozheng Jing, Haiying Yu, Huai Liu

**Affiliations:** 1 College of Plant Protection, Southwest University, Chongqing 400716, China; 15978967939@163.com; 2 Southeast Chongqing Academy of Agricultural Sciences, Chongqing 408000, China; ozzy2311670@163.com (X.Z.); jinghaozheng1988@163.com (H.J.); 3 General Station of Forest and Grassland Pest Management, National Forestry and Grassland Administration, Shenyang 110034, China; yuhaiying2000@126.com

**Keywords:** *Bursaphelenchus xylophilus* determination, nuclear DNA, droplet digital PCR

## Abstract

Pine wilt disease (PWD), which poses a significant risk to pine plantations across the globe, is caused by the pathogenic agent *Bursaphelenchus xylophilus*, also referred to as the pine wood nematode (PWN). A droplet digital PCR (ddPCR) assay was developed for the quick identification of the PWN in order to improve detection sensitivity. The research findings indicate that the ddPCR assay demonstrated significantly higher analysis sensitivity and detection sensitivity in comparison to traditional quantitative PCR (qPCR). However, it had a more limited dynamic range. High specificity was shown by both the ddPCR and qPCR techniques in the diagnosis of the PWN. Assessments of reproducibility revealed that ddPCR had lower coefficients of variation at every template concentration. Inhibition tests showed that ddPCR was less susceptible to inhibitors. There was a strong linear association between standard template measurements obtained using ddPCR and qPCR (Pearson correlation = 0.9317; *p* < 0.001). Likewise, there was strong agreement (Pearson correlation = 0.9348; *p* < 0.001) between ddPCR and qPCR measurements in the evaluation of pine wood samples. Additionally, wood samples from symptomatic (100% versus 86.67%) and asymptomatic (31.43% versus 2.9%) pine trees were diagnosed with greater detection rates using ddPCR. This study’s conclusions highlight the advantages of the ddPCR assay over qPCR for the quantitative detection of the PWN. This method has a lot of potential for ecological research on PWD and use in quarantines.

## 1. Introduction

*Bursaphelenchus xylophilus* (Steiner and Buhrer) Nickle (*B. xylophilus*) is the causative agent of pine wilt disease (PWD), a disease that is extremely damaging to coniferous forests [1,2,3]. It is also known as the pinewood nematode (PWN). Originating in North America, this nematode has been connected to pine wilt in the US, Canada, and Mexico [4,5,6,7]. The PWN was brought to Japan at the beginning of the 20th century [8], and from there it expanded to China [9,10,11]. Despite being included on the A1 list of quarantine pests by the European Plant Protection Organization in July 1985 [12], the PWN was discovered in Portugal in 1999 [13]. Presently, there is no effective method to manage *B. xylophilus*, underscoring the critical importance of preventing the pathogen’s introduction. Currently, the most prevalent detection methods for the PWN involve morphological and molecular biology assays [14,15,16]. However, these methods possess various drawbacks. For instance, morphological assays entail multiple time-consuming steps and necessitate a high level of taxonomical expertise. Furthermore, more than 90 nematode species have been identified in pine wood, some of which bear morphological similarities [17,18]. *Bursaphelenchus mucronatus*, which is widely distributed in Asia and Europe, is non-pathogenic to pines but morphologically resembles *B. xylophilus* [3,19,20]. Both nematodes can be transmitted by native *Monochamus* beetles and occupy the same ecological niche in pine forest ecosystems. The primary morphological distinction between these two species is the shape and size of a mucro on the tail of female *B. mucronatus*. However, female *B. xylophilus* isolates with mucronate tails have been reported in Japan, Portugal, and the United States [7,21,22]. Hence, distinguishing between these two nematode species using morphological techniques is challenging.

Various molecular-detection techniques have been devised for distinguishing *Bursaphelenchus* species, such as internal transcribed spacer restriction fragment length polymorphism (ITS-RFLP) [23,24,25], PCR-based methods with species-specific primers [26,27,28], nested PCR [29,30,31], real-time PCR [32,33,34,35], and loop-mediated isothermal amplification (LAMP) [36,37,38]. These detection approaches are more efficient and rapid compared to morphological assays. Notably, real-time PCR technology is highly sensitive and specific, and it employs closed-tube detection to identify the PWN and other plant pathogens. Nevertheless, routine diagnostic or quarantine scenarios typically interpret most quantitative PCR (qPCR) outcomes qualitatively, only indicating the presence or absence of the PWN. However, quantitative interpretation of qPCR results for the PWN and other pathogenic plant nematodes provides valuable and informative data that are crucial for research endeavors, such as epidemiological investigations, population biology assessments of the PWN, the establishment of biologically significant thresholds, and, particularly, screening for PWN-resistant cultivars. Additionally, diagnostic quantitative methodologies with more feasible and clear-cut thresholds are accessible. For instance, they can determine the presence or absence of the PWN based on a quantitative limit of detection and a false-positive rate.

Using qPCR to quantify nucleic acids is an indirect method that has a number of inherent drawbacks. This method estimates a target DNA’s initial concentration by comparing a sample’s quantification cycle (Cq) value with an external DNA calibrator [39]. A series of known standards, such as positive plasmid DNA containing a target sequence for amplification, are usually measured within the assay’s linear range in order to create the calibrator. However, even when assessing the same specimens, considerable differences in calibrator materials and test performance characteristics can impede agreement between laboratories [40].

Digital PCR (dPCR) is a cutting-edge molecular method that does not require a standard curve and enables a precise measurement of DNA targets. In 1992, Sykes et al. and Vogelstein and Kinzler first presented the idea of digital PCR [41]. Digital PCR has since evolved into droplet digital PCR (ddPCR) and microfluidic chip digital PCR (mcdPCR), which are extensively used in nucleic acid quantification [34]. Target DNA is contained in many nanoliter-sized water-in-oil microdroplets, which are randomly distributed with background DNA, as part of the dPCR technique. This is achieved by partitioning a bulk fluorescence PCR reaction. One or zero copies of the target DNA are contained in each microdroplet, which serves as miniature PCR reactors [42,43]. When the PCR reaction reaches its endpoint, the positive and negative droplets are automatically counted. The percentage of positive droplets and Poisson statistics can be used to calculate the total number of target DNA molecules in the sample. Notably, ddPCR exhibits better accuracy and precision in nucleic acid detection and quantification than qPCR, eliminating the need for an external calibration curve or reference. Thus, it is clear that ddPCR is becoming more and more useful in a variety of fields, including clinical settings [44,45,46], medical research [47], environmental safety [48,49,50], food safety and monitoring [51], and gene-editing studies [52]. It is noteworthy that recent applications of ddPCR include the detection of agricultural pathogens like *Xanthomonas citri* subsp. citri [53] and citrus huanglongbing [54].

In the search for a new technique with higher accuracy and precision, we evaluated the potential for ddPCR to diagnose the PWN. We established a ddPCR approach to detect and quantify the PWN. We then compared this new approach’s linearity, dynamic range, sensitivity, reproducibility, and diagnostic performance with those of qPCR.

## 2. Results

### 2.1. Primers and Probe Design

**Figure 1:**
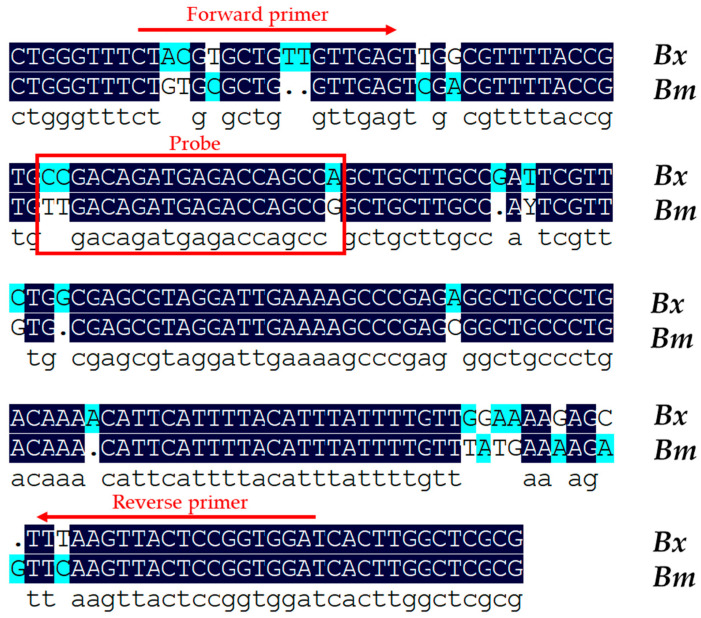
Alignment of the consensus 16SrDNA sequence from the *B. xylophilus* (Bx) and *B. mucronstus* (Bm) isolates. The forward primer and reverse primer are indicated by red arrows, and the probe is indicated by a red box. The consensus DNA sequence from *B. xylupholus* and *B. mucronstus* is highlighted with a dark-blue background. Dots indicate gaps in the corresponding sequence that were added for the alignment.

The *B. xylophilus* 16SrDNA gene was selected as the target gene for the design of a specific set of qPCR and ddPCR primers and a probe. The PCR product exhibited a 171 bp fragment from this DNA sequence. The primers and probe were designed using nucleotide differences between *B. xylophilus* and *B. mucronatus* (Figure 1). The sequences of the primers and probe are presented in Table 1.

### 2.2. Optimization of the ddPCR Assay

**Figure 2:**
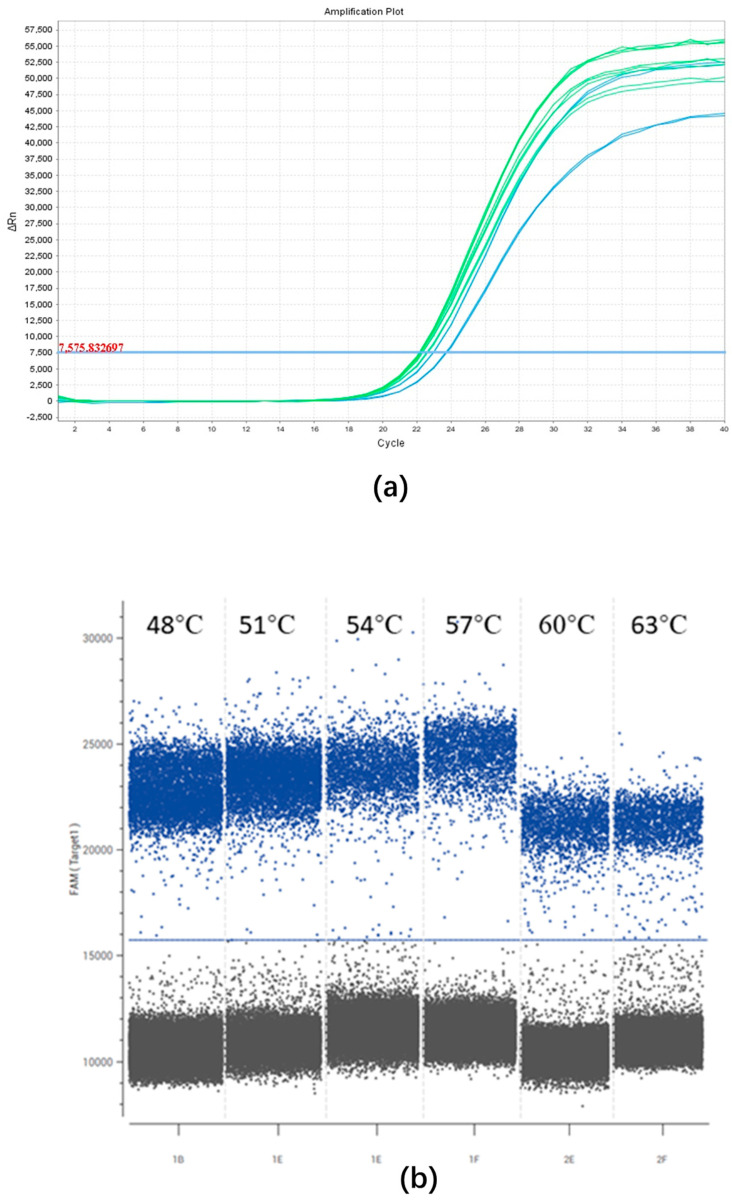
Optimization of the qPCR and ddPCR assays. (**a**) The amplification plot of the qPCR assay displays the optimal extension temperature for the detection of *B. xylophilus*. The unbroken blue line represents the threshold automatically set by the software. The reactions occurred across the following extension temperature gradient: 50 °C, 53 °C, 56 °C, 59 °C, 62 °C, and 65 °C. Different extension temperatures exhibited slight variations in Ct values. (**b**) Fluorescence amplitudes are plotted against an annealing temperature gradient. Positive droplets (blue) with PCR amplification are shown above the unbroken blue line, and negative droplets (black) without any amplification are shown below the line. Six ddPCR reactions, each containing the same number of targets, are separated by a vertical gray dotted line. The reactions occurred across the following annealing temperature gradient: 48 °C, 51 °C, 54 °C, 57 °C, 60 °C, and 63 °C. Different annealing temperatures exhibited slight distinctions in fluorescence between negative and positive droplets, and the highest fluorescence intensity was observed at 57 °C.

To optimize the extension temperature for the qPCR assay, temperatures of 50 °C, 53 °C, 56 °C, 59 °C, 62 °C, and 65 °C were tested. Minor variations in Ct values were observed at the different extension temperatures (Figure 2a), and 59 °C was selected as recommended by the qPCR kit.

To determine the most suitable annealing temperature for the ddPCR assay, temperatures of 48 °C, 51 °C, 54 °C, 57 °C, 60 °C, and 63 °C were tested. Slight variations in fluorescence intensity were observed between negative and positive droplets at the different annealing temperatures, with the highest fluorescence intensity recorded at 57 °C. Consequently, 57 °C was selected for subsequent ddPCR tests (Figure 2b).

### 2.3. Specificity of ddPCR and qPCR

**Figure 3:**
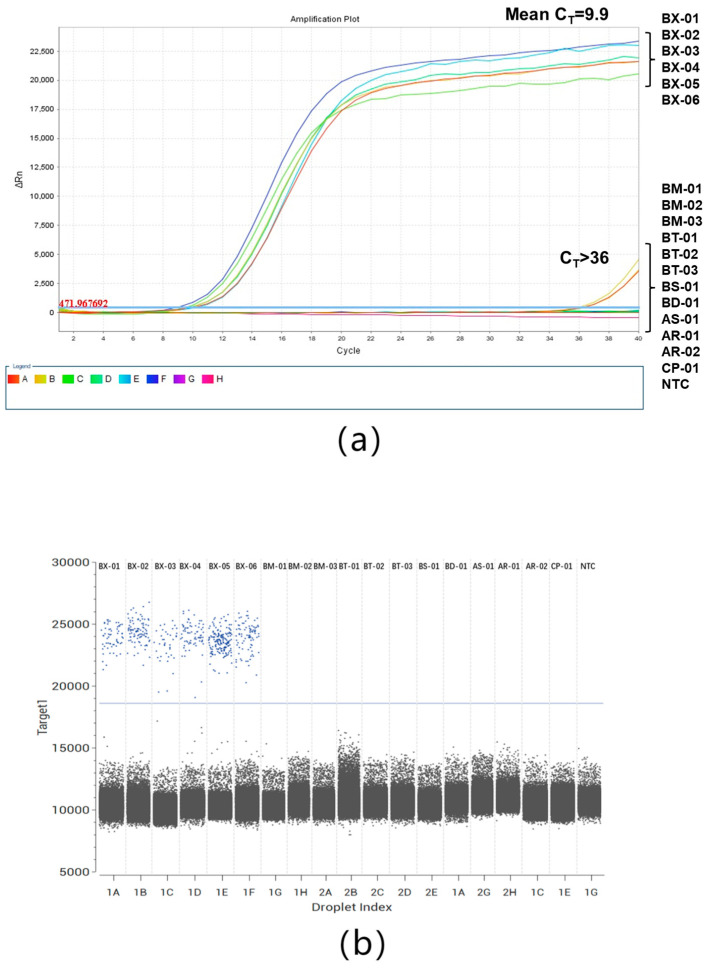
Specificity of qPCR (**a**) and ddPCR (**b**) assays for *B. xylophilus*. Amplification was conducted with DNA templates from *B. xylophilus* (BX01-BX06), *B. mucronatus* (BM01-BM03), *B. thailandae* (BT01-BT03), *B. sexdentati* (BS01), *B. dalianensis* (BD01), *Aphelenchoides parasaprophilus* (AS01), *A. resinosi* (AR01 and AR02), and *Cylindrotylenchus pini* (CP01).

The specificity of the ddPCR and qPCR assays was confirmed using DNA extracted from 6 isolates of *B. xylophilus* (***BX***) and 12 other nematode isolates (Table 2). The DNA samples from all *B. xylophilus* isolates were positive, whereas the DNA samples extracted from the other nematode isolates were negative in the ddPCR and qPCR assays (Figure 3a,b).

### 2.4. Comparison of Analytical Sensitivity, Linearity, and Dynamic Range between ddPCR and qPCR Assays

**Figure 4:**
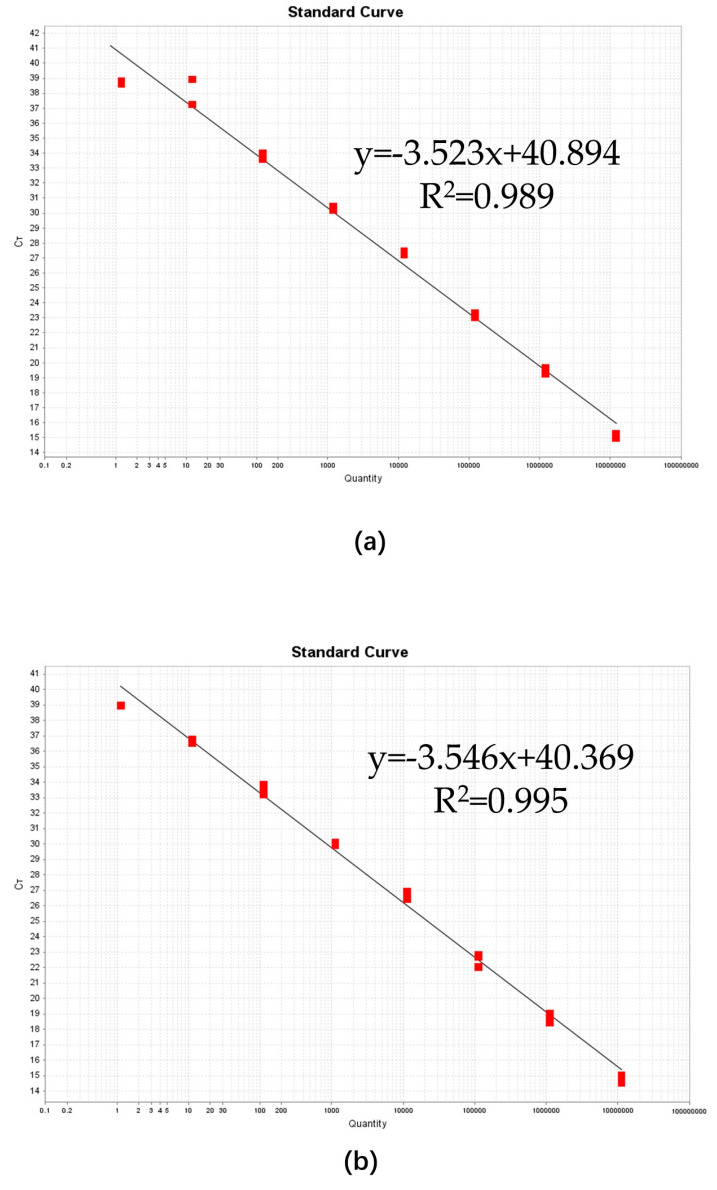
The standard curves of the qPCR assays, which were generated using plasmid DNA (**a**) and samples spiked with pine wood DNA extract (**b**). Plasmid DNA was diluted 10-fold to create eight concentration gradients. The resulting standard curve for the plasmid DNA had a slope of −3.523, corresponding to a PCR efficiency of 92.246% (R^2^ = 0.989). For the spiked samples, the standard curve exhibited a slope of −3.546, which was equivalent to a PCR efficiency of 91.419% (R^2^ = 0.995).

Standard curves were created for the qPCR assay using 10-fold dilutions of positive plasmids (Figure 4a) and positive plasmids spiked with pine wood DNA extract (Figure 4b) to compare the linearity and PCR efficiency between the plasmid samples and the spiked samples. Using positive plasmid DNA (1.20 × 10^7^–1.20 × 10^0^ copies/μL), both tests showed good linearity (R^2^ = 0.989 and R^2^ = 0.995) across the investigated dynamic range. The qPCR standard curves for the positive plasmid DNA possessed a slope of −3.523, indicating a PCR efficiency of 92.246%, while the qPCR curves of the spiked samples had a slope of −3.546, indicating a PCR efficiency of 91.419%. The Ct values and initial logarithmic concentrations of the qPCR serial dilution series are detailed in Table S1.

**Figure 5:**
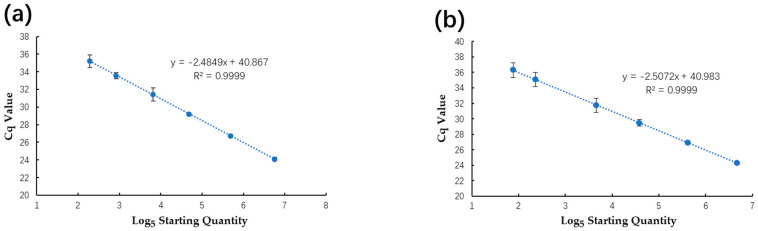
Linear regression of the qPCR assay for plasmid DNA (**a**) and spiked samples (**b**) using the serial dilution series. Both of these two assays exhibited excellent linearity (both R^2^ = 0.999).

Regression curves were established for the qPCR (Figure 5) using a series of 5-fold dilutions of positive plasmids and spiked samples to compare the analytical sensitivity, linearity, and dynamic range between the qPCR and ddPCR. Using positive plasmid DNA (1.32 × 10^4^–0.84 × 10^−1^), the qPCR tests for both plasmid DNA and spiked samples showed excellent linearity, with corresponding R^2^ values of 0.999 across the investigated dynamic range. The qPCR tests’ calculated sensitivities for the plasmid DNA and spiked samples were estimated to be 18.67 copies/μL and 34.35 copies/μL, respectively. The qPCR serial dilution series’ Cq values and initial logarithmic concentrations are detailed in Table S2.

**Figure 6:**
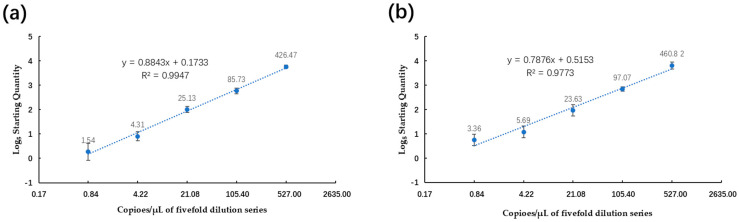
Linear regression of the ddPCR assay for plasmid DNA (**a**) and spiked samples (**b**) constructed using the same serial dilution series tested with the qPCR assay (see Figure 5). The estimated Pearson correlation coefficient of the plasmid DNA regression curve (y = 0.8843x + 0.1733) is 0.9947 (R^2^ = 0.9991, *p* < 0.0001). The estimated Pearson correlation coefficient of the regression curve of the spiked samples (y = 0.7876x + 0.5153) is 0.9773 (R^2^ = 0.9773, *p* < 0.0001). The standards tested using ddPCR exhibited a dynamic range of five orders of magnitude. The vertical axis shows the log5-transformed copy number/μL of the ddPCR reaction mixture. The horizontal ordinate indicates the expected log5-transformed concentration of the copy number/μL of the ddPCR reaction mixture.

**Figure 7:**
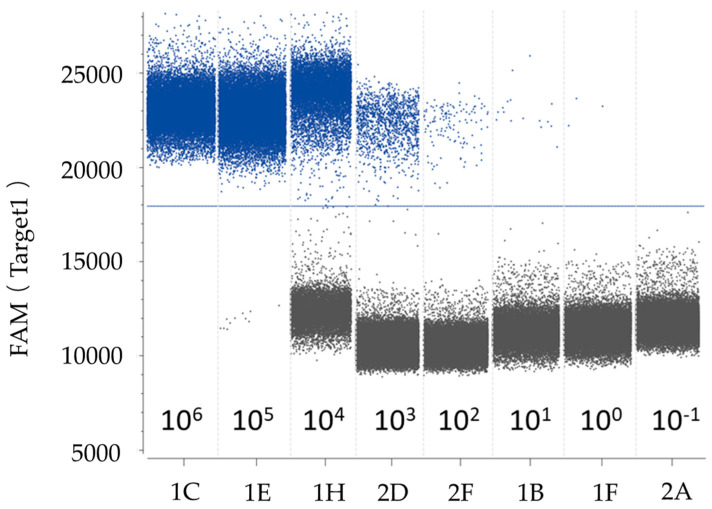
A representative 1-D plot of ddPCR reactions. The ordinate indicates the fluorescence amplitude, and the blue line is the threshold. Above the threshold are positive droplets (blue) containing at least one copy of the target DNA, and below the threshold are negative droplets (gray) without the target DNA. Eight ddPCR reactions with various serially diluted targets are divided by the vertical dotted line. When the target DNA exceeded 10^6^, the ddPCR reaction was saturated by an excess target DNA concentration, and when the target DNA was 10^−1^, the ddPCR reaction provided a negative result.

The plasmid DNA standards and spiked samples were also employed to evaluate the quantitative linearity of the ddPCR assay. The copy numbers of serially diluted plasmid DNA, as determined via ddPCR, were plotted against log5-transformed values and fitted with a linear regression model. The ddPCR assay measurements indicated that both the positive plasmid standards and spiked samples (Figure 6) displayed strong linearity (R^2^ = 0.9947, R^2^ = 0.9773, *p* < 0.0001). Notably, it was observed that at a target concentration of 106 copies/μL, droplets exhibited positive saturation, rendering the Poisson algorithm invalid and resulting in a narrow dynamic range in comparison to qPCR. Remarkably, in this investigation, the sensitivity values of the ddPCR assay for the positive plasmid standards and spiked samples were as low as 1.54 copies/μL and 3.36 copies/μL, indicating it had greater sensitivity than qPCR. A representative one-dimensional plot of ddPCR reactions with serially diluted targets is depicted in Figure 7. The copy numbers of the serial dilution series detected using the ddPCR as-say are provided in Table S3.

### 2.5. Comparison of Reproducibility between qPCR and ddPCR Assays

**Figure 8:**
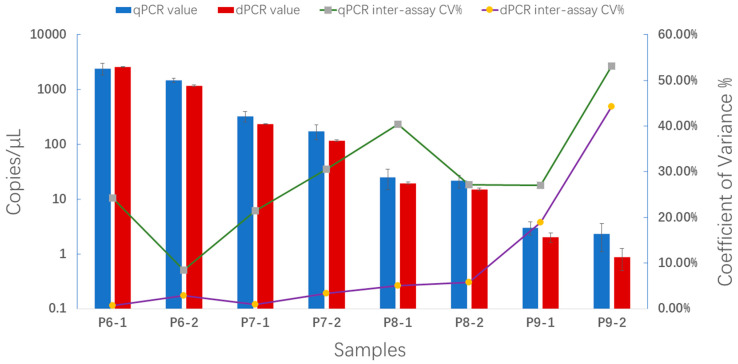
The inter-assay CV% of the qPCR and ddPCR assays. Samples P6-1, P6-2, P7-1, P7-2, P8-1, P8-2, P9-1, and P9-2 were positive plasmid DNA; among them, P6-1, P6-2, P7-1, and P7-2 had high concentrations, while P8-1, P8-2, P9-1, and P9-2 had low concentrations. Histograms indicate the average copy number of each sample on a log10 scale. Lines show the CV variation trends of the qPCR and ddPCR assays during repeated tests with diverse sample concentrations. The ddPCR assay was more precise than the qPCR assay for quantification of the PWN, especially for low target concentrations (numerical data supporting Figure 6 are provided in Table S4).

Eight positive plasmid DNA samples were utilized to assess the reproducibility of qPCR and ddPCR. As depicted in Figure 8, ddPCR demonstrated superior reproducibility in comparison to qPCR (CV% decreased by 97.4–16.5%; numerical data that support Figure 8 are provided in Table S3), indicating that fewer repetitions were acceptable in this experiment.

Four different volumes (0, 10%, 20%, 30%, and 40%) of pinewood extracts (which contain inhibitors) were added to reaction systems to evaluate their effect on the reproducibility of the qPCR and ddPCR assays. The results showed that the inhibitors had no influence on PCR reproducibility. There were no correlations between CV% and the inhibitors at the various percentages (R^2^ = 0.6729 for qPCR and R^2^ = 0.2563 for ddPCR). More detail is presented in Figure S1.

### 2.6. The Influence of PCR Inhibitors on the qPCR and ddPCR Assays

**Figure 9:**
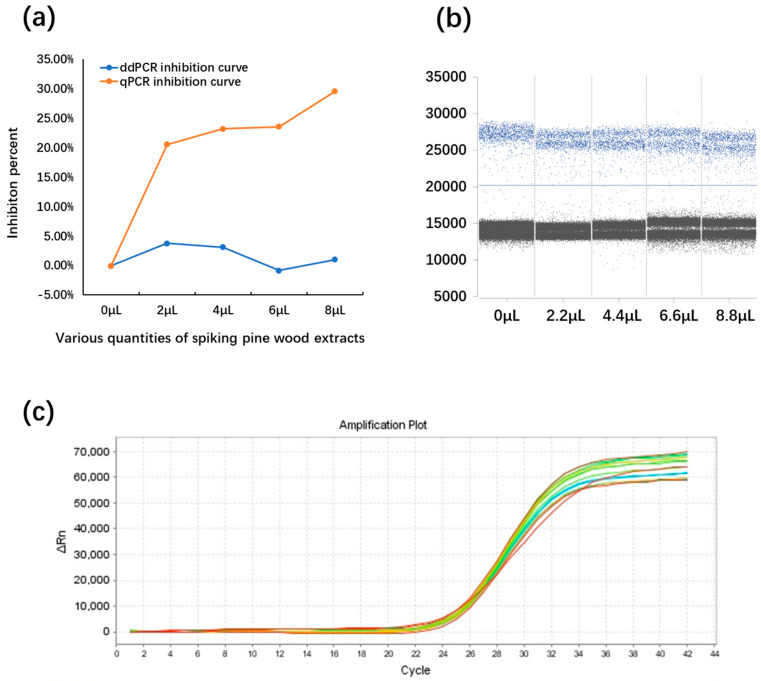
The influence of samples spiked with different volumes of pine wood extracts. ddPCR exhibited superior tolerance to pine wood extracts compared to the qPCR assay. qPCR was more susceptible to the inhibitors (**a**). In the ddPCR assays, the fluorescence intensity of positive droplets decreased slightly with an increase in the inhibitor volume (**b**). In the qPCR assays, the fluorescence curves exhibited no significant differences between different volumes of inhibitors (**c**) (numerical data supporting Figure 9 are provided in Table S5).

The resilience of the qPCR and ddPCR assays was estimated with reactions containing different volumes of pine wood extracts. The reactions were spiked with the same concentrations of PWN genomic DNA, and resilience was determined based on the ratios between the target concentrations in the presence of different volumes of inhibitors and in the absence of inhibitors (Figure 9). In the inhibition assay, the qPCR suppression curve was above that of ddPCR, indicating that the qPCR assay was more susceptible to the inhibitors (Figure 9a). Interestingly, the ddPCR assay showed decreased fluorescent signals for positive droplets (Figure 9b). However, in the ddPCR assay, even with an inhibitor ratio of up to 40%, the positive and negative droplets could still be distinguished clearly. For qPCR, the fluorescence curves of the inhibition assay exhibited no significant differences between different concentrations of inhibitors, while the Ct values exhibited subtle changes (Table S5).

### 2.7. Comparison of Detection Sensitivity between ddPCR and qPCR Assays

A total of 37 nematode-free wood samples spiked with single PWNs were assessed to compare the detection sensitivity between the ddPCR and qPCR assays. All wood samples spiked with single PWNs tested positive when using ddPCR. Correspondingly, 26 out of 37 (72.27%) wood samples were detected using qPCR, which indicated that ddPCR had a higher detection rate than qPCR (numerical data from the ddPCR and qPCR assays are provided in Table S6).

### 2.8. Evaluation of the Method for Extracting DNA from Wood Samples

Five DNA extracts recovered from single PWNs from pure cultures and five DNA extracts recovered from nematode-free pine wood samples spiked with single PWNs were used to evaluate the DNA recovery efficiency. The results showed that all DNA samples tested positive when using ddPCR, five DNA extracts from PWNs from pure cultures tested positive using qPCR, and three out of five spiked wood samples tested positive using qPCR. We also found that the DNA samples from PWNs from pure cultures exhibited lower Ct values (30.91 versus 34.30) when using qPCR assays and exhibited higher concentrations (14.39 copies/μL versus 1.76 copies/μL) when using ddPCR assays. These results indicate that extracting DNA from the spiked wood samples resulted in a loss of most of the genomic PWN DNA (numerical data from the ddPCR and qPCR assays are provided in Table S7).

### 2.9. Comparison of Diagnostic Performance between ddPCR and qPCR Assays

**Figure 10:**
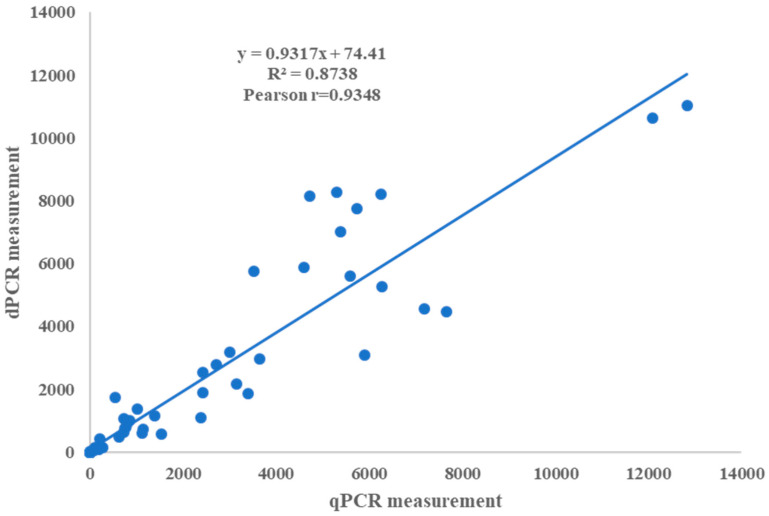
Correction of qPCR and ddPCR measurements. Measurements of pine wood samples using qPCR and ddPCR assays were significantly correlated (Pearson r = 0.9348, *p* < 0.0001). Solid line indicates fitting curve (numerical data from ddPCR and qPCR assays are provided in Table S8).

A total of 87 wood samples from pine trees, comprising 37 from deceased pine trees, 15 from symptomatic trees, and 35 from asymptomatic trees, were subjected to analysis using both the ddPCR and qPCR assays to evaluate the potential replacement of the latter by the former. The correlation between the PWN measurements obtained with the ddPCR and qPCR assays demonstrated a robust linear association with a Pearson’s correlation coefficient of R^2^ = 0.8751 (*p* < 0.001). Notably, the linear model revealed a significant slope and intercept for the PWNs identified via ddPCR and qPCR (*p* < 0.0001), with respective values of 0.9317 and 74.41 (Figure 10). PWNs were detected in 62 out of 87 (71.62%) pine wood samples from epidemic areas, including 97.3% from deceased pine trees, 100% from symptomatic trees, and 31.4% from asymptomatic trees. Correspondingly, 50 out of 87 (57.47%) wood samples tested positive using qPCR, including 97.3% from deceased trees, 86.67% from symptomatic trees, and 2.9% from asymptomatic trees (Table 3). Importantly, the overlap between the PWN-positive wood samples detected via ddPCR and qPCR was not complete, with 65 out of 87 samples (74.71%) demonstrating concordance between the two methods (Table 4). Notably, six samples with Ct > 35 that were deemed PWN-negative using qPCR were found to be positive using ddPCR. These findings underscore the superior positive predictive value and heightened sensitivity of the ddPCR assay compared to qPCR, particularly for samples in the latent or asymptomatic phases of infection.

## 3. Discussion

*Bursaphelenchus xylophilus*, the pine wood nematode (PWN), the causative agent of pine wilt disease (PWD), has caused extensive devastation to pine trees and forests over the past century [55,56]. It has been documented that long-distance dispersal of the PWN is facilitated by human activities, particularly the transportation of wood [3,11,55]. Rapid and accurate detection of the PWN in these products is critical to prevent PWN introduction [16,34,57,58]. Presently, real-time quantitative PCR (qPCR) is utilized to diagnose the PWN, providing sensitive, reliable, and rapid detection and identification of the PWN, as well as other plant pathogens [33,35,59,60,61,62]. However, qPCR is insufficient for quantitative analysis due to its reliance on the relationship between the cycle quantification (Cq) value of a test sample and a calibration curve. Cq values are influenced by thresholds and PCR efficiency, with the determination of thresholds typically based on empirical knowledge and PCR efficiency being susceptible to variations across different reactions. Consequently, a sample may yield differing quantitative outcomes in different runs or even within the same run. In experiments involving absolute quantification, an external calibration curve is necessary for each plate, even when analyzing only one sample, making this process costly, labor-intensive, and time-consuming. Furthermore, the reliability of standard solutions can also impact quantification via qPCR. For instance, the degradation of reference DNA materials or differences in handling conditions can lead to quantitative results that fluctuate on a daily basis, both within a laboratory and across different laboratories.

In contrast to conventional qPCR, the recently developed droplet digital PCR (ddPCR) technology offers numerous advantages, including enhanced sensitivity; absolute quantification without the requirement for an external calibration curve; reduced susceptibility to PCR inhibitors and variations in PCR efficiency; augmented precision, particularly in samples with low concentrations; improved accuracy, reliability, and between-run reproducibility; and a heightened capability to discern small changes in concentrations [63,64,65,66,67,68,69]. In our investigation, we formulated a *B. xylophilus* detection approach utilizing qPCR and ddPCR assays and compared their linearity, sensitivity, and reproducibility to assess the potential utility of ddPCR for quantitative detection of the PWN. Both ddPCR and qPCR exhibited satisfactory linearity, and a strong correlation was observed between the ddPCR and qPCR measurements (R^2^ = 0.8738, *p* < 0.0001). These findings are in line with those reported by Zhao et al. and Zhong et al. [53,54]. However, in comparison to qPCR, ddPCR demonstrated a limited dynamic range when the target concentrations were at 10^6^ copies/L and the droplets were positively saturated, rendering the Poisson algorithm invalid. Consequently, sample dilution is necessary when the target concentration is high. Nonetheless, ddPCR displayed a lower detection limit, suggesting that it is more sensitive than qPCR.

Upon comparing the reproducibility of the two methods, we observed that both ddPCR and qPCR demonstrated favorable reproducibility at high target concentrations. However, at low target concentrations, the ddPCR assay exhibited more consistent quantitative outcomes in comparison to the qPCR assay. Thus, ddPCR proved to be more sensitive and reproducible than qPCR, which aligned with previous studies [53,54,64,68]. Wood samples, which encompass intricate constituents, are known to contain PCR inhibitors [44,70]. Co-extracted phenolic compounds commonly found in wood have been proven to be the main inhibitory components [70,71]. To mitigate the potential impact of PCR inhibitors, we employed microscopy and the Baermann extraction procedure to extract nematodes from wood samples prior to DNA extraction. This was the first study to use this method (Cao et al. and Leal et al.) [28,32,33]. These procedures are labor-intensive and time-consuming. In contrast, ddPCR exhibits reduced susceptibility to PCR inhibitors compared to qPCR, permitting the use of the ddPCR assay to extract total DNA from wood samples as a template [53,66,67]. In our study, pine wood extracts were used as inhibitors to evaluate their inhibitory effects during ddPCR and qPCR. We found that ddPCR is less susceptible to the inhibitors. Therefore, we extracted total DNA from 87 wood samples and used it as templates in the ddPCR and qPCR assays. Consequently, ddPCR proved to be a more suitable method for the detection of *B. xylophilus* in field wood samples with complex inhibitor matrices.

The qPCR assay employed a conservative detection threshold of Ct = 35 for positive/negative diagnostics in the detection of *B. xylophilus* in wood samples as per a study conducted by Leal et al. [33]. This threshold was based on a mean cycle threshold value of Ct = 33.75 (±0.77), which was derived from amplification of single nematodes. However, due to potential DNA loss during extraction and the limitation of testing only 8 mL of the DNA extraction solution, the Ct value may exceed 35 for single nematodes in wood samples. Raising this threshold may result in false positives. In contrast, ddPCR directly detects negatives and positives and exhibits higher sensitivity, allowing for the easy detection of single nematodes in wood samples. However, in our study, we directly extracted total DNA from wood samples using a DNA extraction kit. This approach offers more convenience and efficiency compared to the methods of Cao et al. and Leal et al. [32,33]. Additionally, the presence of tiny wood chips in the crude extract may obstruct the capillary during droplet generation. Therefore, sedimentation of the crude extract using ethyl alcohol and purification via an adsorption column are necessary.

In our study, a commercial kit was used to extract total DNA from wood samples. In previous studies, various methods were used to extract total DNA from wood samples, and the extracts were used for nested PCR [29,31], LAMP [36,37,38], and qPCR [33,35,59,72] to detect PWNs. However, these extracts contained tiny wood chips that could block the cartridge during droplet generation. Unfortunately, the recovery rate of the PWN genomic DNA was very low in our study, which may have resulted in false negatives in the qPCR assay. However, there was no such problem in the ddPCR assay due to its high sensitivity.

Following the decay of an organism, the half life of DNA in natural conditions is estimated to be approximately 521 years [73]. Existing molecular-detection methods that use DNA as a template are unable to differentiate between live and dead nematodes. Therefore, in our ongoing investigations, we aim to utilize ddPCR to detect mRNA, integrating reverse transcription and PCR in a single-step operation to enhance the convenience and accuracy of detection and quantification. This study presents the development of a specific PWN-detection system, which is the first to incorporate ddPCR technology to quantify PWNs. We conducted a comparative assessment of linearity, sensitivity, the coefficient of variation (CV), and the relevance ratio between the ddPCR and qPCR assays. The results indicate that ddPCR is a dependable alternative method for quantitatively detecting the PWN. The implications of these findings extend beyond PWN quarantine measures, offering a robust tool for studying infestations and diffusion patterns of the PWN. As the PWN is a multicellular organism, further investigations are warranted to quantify PWNs in wood samples.

## 4. Materials and Methods

### 4.1. Nematodes, PWN-Infected Pinewood Samples, and Spiked Samples

Pure cultures of six *B. xylophilus* isolates were obtained from pinewood samples collected in Liaoning, Chongqing, Shandong, Zhejiang, Guangdong, and Fujian, China. In addition, three isolates of *B. mucronatus*, three isolates of *B. thailandae*, one isolate of *B. sexdentati*, one isolate of *B. dalianensis*, one isolate of *Aphelenchoides parasaprophilus*, two isolates of *A. resinosi*, and one isolate of *Cylindrotylenchus pini* were used to examine the specificity of the qPCR and ddPCR assays. Details of the nematodes used in this study are presented in Table 2. In this study, all nematode isolates were cultured on potato dextrose agar (PDA) plates colonized by the fungus *Botrytis cinerea*.

To prepare wood samples, approximately 1 g of wood was collected from 87 trees using a drill with a diameter of 1.0 cm. The sampled pine trees comprised 37 deceased trees, 15 symptomatic trees, and 35 asymptomatic trees. The asymptomatic trees that were sampled were found to have fresh feeding trails made by *Monochamus alternatus*. In a follow-up survey after 1–2 months, the symptomatic trees were confirmed via positive samples, and the trees that were still asymptomatic were excluded.

Spiked wood samples used to simulate the presence of wood tissue were prepared by mixing one adult *B. xylophilus* with 50 mg of nematode-free wood tissue from a pine tree. Total DNA was extracted from this mixture for a sensitivity assay. Additionally, total DNA was extracted from 50 mg of nematode-free wood tissue from a pine tree, and the extraction solution was added to the reaction mixture for qPCR and ddPCR to simulate the presence of wood tissue and to compare the analytical sensitivity, linearity, and dynamic range between the ddPCR and qPCR assays.

Field wood samples were collected from infected (confirmed previously) and asymptomatic pine trees growing in the Fuling district of the Chongqing municipality of China. Nematode isolates were supplied by the General Station of Forest and Grassland Pest Management of the National Forestry and Grassland Administration. All samples were safely disposed of after collection, and there were no protected species growing in the sample area.

### 4.2. DNA Extraction

Pure cultures of nematodes were collected from PDA plates and washed twice with double-distilled water. The nematodes were flash-frozen in liquid nitrogen for 1 min and incubated at 65 °C for 3 min in order to disrupt their cells. This step was repeated three times. The total DNA of the nematode suspensions was extracted using a commercial mollusk DNA kit (Omega, Norcross, GA, USA). When DNA was extracted from the wood samples and nematode-free wood samples, a Universal Genomic DNA Extraction Kit (Solarbio, Beijing, China) was used. The total DNA from the nematodes from the pure cultures was extracted using the method of Cao et al. [32].

DNA was extracted from the wood samples and nematode-free wood samples according to the following steps: (1) Weigh 50 mg of a wood sample in a 2 mL centrifuge tube, freeze it using liquid nitrogen, and grind it using a JXFSTPRP64 automatic grinding apparatus (Jingxin, Shanghai, China). (2) Add 500 μL of Solution A (Solarbio, Beijing, China) and vortex to mix thoroughly. (3) Add 20 μL of RNase A (10 mg/mL) to the suspension and incubate at 55 °C for 10 min. (4) Add 20 μL of proteinase K (10 mg/mL) to the suspension, incubate at 55 °C for 30 min, centrifuge at 12,000 rpm for 10 min, and transfer the clear supernatant into a new tube. (5) Add 500 μL of Solution B and vortex to mix thoroughly. (6) Add 500 μL of ethanol, vortex to mix thoroughly, transfer the suspension into the column, and reuse the collection tube. (7) Centrifuge at 12,000 rpm for 1 min, discard the filtrate, and reuse the collection tube. (8) Add 600 μL of wash solution, centrifuge at 12,000 rpm for 1 min, discard the filtrate, and reuse the collection tube. (9) Repeat step (8). (10) Centrifuge at 1200 rpm for 2 min and allow the sample to stand at room temperature or 55 °C for several minutes. (11) Transfer the column to a clean 1.5 mL microcentrifuge tube. (12) Add 50–200 μL of elution buffer directly to the center of the column membrane, allow it to stand at room temperature for 5 min, then centrifuge at 12,000 rpm for 2 min. The resulting filtrate contains the total DNA extracted from the wood sample.

### 4.3. Primers and Taqman Probe Design

The genus-specific primers P1663F and P2562R (Table 1) were used to amplify a part of the 16srDNA region of *Bursaphelenchus* spp (Genebank sequence ID: MK 292122.1). PCR products from six isolates of *B. xylophilus* and three isolates of *B. mucronatus* were cloned and sequenced. The sequences were aligned, and specific primers and Taqman^®^ probes were designed for these sequences using online software (https://www.genscript.com/tools/real-time-pcr-taqman-primer-design-tool (accessed on 12 February 2021). The Taqman^®^ probe was dual-labeled at the 5′ end with fluorescent reporter dye (6-carboxy-fluorescein, FAM) and at the 3′ end with 5-carboxy tetramethylrhodamine (TAMRA). The sequences of the forward and reverse primers were named P1904-F and P2052-R, respectively. The probe was named Probe-1938.

### 4.4. Preparation of Cloned Plasmid Standard

A 16srDNA segment of *B. xylophilus* was amplified using the primers P1663F and P2562R as described below with PWN genomic DNA as the template. The PCR amplicon was purified using an Omega gel extraction kit (Omega, Norcross, GA, USA) and ligated into a pMD19-T cloning vector (TaKaRa, Shiga, Japan). The ligation products were transformed into competent *Escherichia coli* JM109 cells (TransGen Biotech, Beijing, China). A single positive colony was cultured in a liquid LB medium at 37 °C with shaking at 150 rpm for 10–15 h, and the plasmid was extracted using an Omega E.Z.N.A.^®^ Plasmid Mini Kit I (Omega, USA). The plasmid DNA was linearized via the endogenous restriction enzyme.

EcoRI (TaKaRa, Shiga, Japan). The copy number of the linearized plasmid DNA was quantified using ddPCR (Sinper, Suzhou, Jiangsu, China) and recalculated to determine the plasmid copies/L. The linearized plasmid DNA was diluted 10-fold into nine concentration gradients, and the diluents were used to test the analytical sensitivity, linearity, and dynamic range of qPCR and ddPCR.

### 4.5. Quantitative PCR

qPCR assays using the Applied Biosystems SteponePlus Real-Time PCR System (LifeTech, Gaithersburg, MD, USA) used 20 L of reaction mixtures containing TaqPro™ HS Universal U+ Probe Master Mix (2; Vazyme, Nanjing, Jiangsu, China), 400 nmolL^−1^ of each primer, 200 nmol L^−1^ of the probe (as recommended for this qPCR kit), a 5 L sample of the template, and 4 L of double-distilled water. The qPCR thermocycling conditions included digestion of contamination at 37 °C for 2 min, initial denaturation at 95 °C for 5 min, and 40 cycles of 95 °C for 10 s and 59 °C for 30 s. The Cq values were analyzed using StepOne Software V2.3 with autocalculated baseline settings and a manually set threshold of 0.1. Standard curves constructed for serial dilutions of plasmid DNA were included in every qPCR run to produce quantitative results rather than raw Cq values.

The optimal extension temperature was determined by performing qPCR at 50 °C, 53 °C, 56 °C, 59 °C, 62 °C, and 65 °C. An extension temperature was considered optimal if it produced the smallest Ct values and maximized fluorescence.

### 4.6. Droplet Digital PCR

The DQ24™ Droplet Digital™ PCR system (Sinper, Suzhou, Jiangsu, China) was used in this study. The total volume for ddPCR was 22 L, which contained 11 L of ddPCR Master Mix (2×) (Sinper, Suzhou, Jiangsu, China), 800 μmol L^−1^ of each primer, 400 μmol L^−1^ of the probe, and 5.5 μL of sample DNA. Droplet generation, PCR reactions, and droplet counting were performed using a digital PCR system with one-step operation. The protocol for the PCR reaction was as follows (temperature ramp rate: 2 °C S^−1^): 60 °C for 5 min, 95 °C for 15 min, 45 cycles of 94 °C for 30 s, and annealing at 57 °C for 20 s, and finally 60 °C for 1 min. After the PCR reaction, the droplets were automatically counted using the digital PCR system.

The optimal annealing temperature for the ddPCR assay was established by testing at temperatures ranging from 48 °C to 63 °C. The temperature that yielded the greatest distinction in fluorescence between negative and positive droplets was deemed optimal.

### 4.7. Assessing Inter-Assay Variability between ddPCR and qPCR Assays for Independent Experiments

To evaluate the reproducibility of the qPCR and ddPCR assays, independent replicate experiments were performed in triplicate using eight plasmid DNA samples. The eight samples were assayed in three different runs on both platforms. The coefficient of variation (CV) was calculated from the triplicate qPCR and ddPCR assays to reflect the reproducibility between the different runs.

### 4.8. Estimation of Tolerance to Inhibitors

We assessed the tolerance of the qPCR and ddPCR assays to inhibitors present in the wood samples by incorporating varying volumes of pine wood extracts into the reactions. Each reaction was spiked with an equal amount of *B. xylophilus* DNA, and resilience was determined by calculating the ratios between the target concentrations in the presence of different volumes of inhibitors and that in their absence (the negative control for inhibition consisted of double-distilled water supplemented with *B. xylophilus* DNA). To prepare pine wood extracts, 10 g of healthy pine wood were cut into small pieces, soaked in double-distilled water, and incubated at 25 °C overnight. The mixture was then filtered through a 0.45 μm filter, and the filtrate was collected to test the tolerance of both the qPCR and ddPCR assays to the pine wood extracts.

### 4.9. Data Analysis

The qPCR Cq values and standard curves were generated using StepOne Software version 2.3 (Lifescience, Gaithersburg, MD, USA). A linear regression of the qPCR standard curves was recalculated using StepOne Software version 2.3 (Lifescience, Gaithersburg, MD, USA). The slope of the standard curve was used to determine the PCR efficiencies. For ddPCR, the fluorescence amplitude threshold used to discriminate positive and negative droplets was manipulated using the software SightPro version 0.3.12 (Sinper, Suzhou, Jiangsu, China). The copy concentration of each sample was generated using SightPro™ software. To determine the linear range of the ddPCR assay, serial dilution of linearized plasmid DNA was used for ddPCR measurements and comparison with expected values. *t*-tests were used to compare measurement differences for the qPCR and ddPCR assays between the healthy and infected groups. Statistical analyses were performed using Microsoft Excel software 2019MSO (version 2408 Build 16.0.17928.20114) (Microsoft, Washington, DC, USA). Pearson’s correlations and linear regression were also used to evaluate the relationships between the qPCR and ddPCR assay measurements.

## Figures and Tables

**Table 1:** Primers and probes used in this study.

Primer and Probe Name	Primer and Probe Sequence 5′-3′	TM	%GC	Length
P1663F	GATGTTTCGGCATTGTCTTT	53.9	40	20
P2562R	GCTTACTGATATGCTTAAG	41.4	37	19
P1904-F	CTACGTGCTGTTGTTGAG	46.4	50	18
P2052-R	TCCACCGGAGTAACTTAA	47.4	44	18
Probe-1938	5′-FAM-CCGACAGATGAGACCAGCCA-TAMRA-3′	60.8	60	20

**Table 2:** Isolates and origins of nematodes used in this study.

Serial Number	Latin Name	Sample Name	Origin
1	*Bursaphelenchus xylophilus*	BX-01	Liaoning
2	*Bursaphelenchus xylophilus*	BX-02	Chongqing
3	*Bursaphelenchus xylophilus*	BX-03	Shandong
4	*Bursaphelenchus xylophilus*	BX-04	Zhejiang
5	*Bursaphelenchus xylophilus*	BX-05	Guangdong
6	*Bursaphelenchus xylophilus*	BX-06	Fujian
7	*Bursaphelenchus mucronatus*	BM-01	Jilin
8	*Bursaphelenchus mucronatus*	BM-02	Neimenggu
9	*Bursaphelenchus mucronatus*	BM-03	Heilongjiang
10	*Bursaphelenchus thailandae*	BT-01	Shandong
11	*Bursaphelenchus thailandae*	BT-02	Liaoning
12	*Bursaphelenchus thailandae*	BT-03	Jilin
13	*Bursaphelenchus sexdentati*	BS-01	Jilin
14	*Bursaphelenchus dalianensis*	BD-01	Liaoning
15	*Aphelenchoides parasaprophilus*	AS-01	Guangdong
16	*Aphelenchoides resinosi*	AR-01	Liaoning
17	*Aphelenchoides resinosi*	AR-02	Jilin
18	*Cylindrotylenchus pini*	CP-01	Hunan

**Table 3:** Performance of ddPCR and qPCR assays for detection of PWNs in dead, symptomatic, and asymptomatic pine trees.

Pine Wood Sample	ddPCR		qPCR	
	Positive	Negative	Positive	Negative
Dead pine trees	36	1	36	1
Symptom pine trees	15	0	13	2
Asymptomatic pine trees	11	24	1	34

**Table 4:** Correlation of pine wood samples between ddPCR and qPCR assay.

Pine Wood Sample by qPCR	Pine Wood Sample by ddPCR	
	Positive	Negative
Positive	50	0
Negative	12	5

## Data Availability

Data are contained within the article or Supplementary Material.

## Supplementary Materials

The following supporting information can be downloaded at https://www.mdpi.com/article/10.3390/plants13192701/s1, Table S1: Cq values of qPCR serial dilution series for the standard curve; Table S2: Cq values of qPCR serial dilution series; Table S3: Copy numbers of dPCR serial dilution series; Table S4: Quantitative results of qPCR and dPCR methods for reproducibility comparison; Table S5: Quantitative results of qPCR and ddPCR methods for inhibitor comparison; Table S6: Quantitative results of qPCR and ddPCR methods for inhibitor comparison; Table S7: Quantitative results of qPCR and ddPCR methods for different extraction methods; Table S8: Diagnostic results of qPCR and ddPCR for detecting wood samples; Figure S1: Linear regression of the CV% and inhibitor percentage for the qPCR and ddPCR assays.
