# Supplementary material for: Establishment of a Sensitive and Reliable Droplet Digital PCR Assay for the Detection of Bursaphelenchus xylophilus
Source: Plants (Basel). 2024 Sep 26;13(19):2701. doi: 10.3390/plants13192701 (PMC11478728; doi:10.3390/plants13192701)
Supplement: Supplementary file 1 [file plants-13-02701-s001.zip › plants-3157087-supplementary.pdf]

Table S1 Cq values of qPCR serial dilution series for standard curve

Plasmid DNA[a] (copies/μL)	Positive plasmid DNA		Spiked samples	
	Ct value	Ct means	Ct value	Ct means
1.20E+7	14.94	14.92	14.54	14.78
	15.05		14.81	
	14.77		15.00	
1.20E+6	19.52	19.28	18.42	18.73
	19.07		18.73	
	19.26		19.04	
1.20E+5	23.15	23.01	22.03	22.51
	22.89		22.68	
	22.97		22.81	
1.20E+4	27.02	27.12	26.42	26.67
	27.25		26.66	
	27.08		26.93	
1.20E+3	30.20	30.16	29.90	30.01
	30.02		30.08	
	30.26		30.06	
1.20E+2	33.57	33.59	33.46	33.51
	33.79		33.86	
	33.41		33.21	
1.20E+1	37.08	37.91	36.57	36.61
	38.73		36.76	
	Undetermined		36.51	
1.20E+0	Undetermined	38.55	Undetermined	38.97
	38.64		38.96	
	38.46		38.97	
NTC[b]	N/A		N/A	

a. Values reflect copies/μL of calculated serial dilution of positive plasmid DNA standard.
b. NTC means no template control.

Table S2 Cq values of qPCR serial dilution series

Plasmid DNA[a] (copies/μL)	Positive plasmid DNA		Spiked samples	
	Ct value	Ct means	Ct value	Ct means
6.59E+4	24.00	24.09	24.24	24.28
	24.33		24.18	
	23.95		24.41	
1.31E+4	26.92	26.71	27.11	26.89
	26.54		26.72	
	26.66		26.83	
2.64E+3	29.31	29.19	29.40	29.47
	29.06		29.90	
	29.18		29.10	
5.27E+2	30.67	31.44	32.04	31.72
	31.50		31.41	
	32.14		31.72	
1.05 E+2	33.15	33.56	36.08	35.08
	33.83		34.91	
	33.70		34.26	
2.10E+1	35.31	35.20	36.52	36.29
	34.44		35.23	
	35.84		37.13	
NTC[b]	N/A		N/A	

a. Values reflect copies/μL of calculated serial dilution of positive plasmid DNA standard.

b. NTC means no template control.

Table S3 Copy number of dPCR serial dilution series

Plasmid DNA[a] (copies/μL)	Positive plasmid DNA		Spiked samples	
	Copy number	Mean	Copy number	Mean
5.27E+2	392.47	426.46	428.52	460.86
	408.35		370.23	
	478.78		583.75	
1.05 E+2	103.45	85.73	112.36	97.06
	74.63		86.48	
	79.23		92.57	
2.10E+1	28.34	25.13	31.46	23.63
	19.62		15.23	
	27.51		24.32	
4.22E+0	5.13	4.31	6.38	5.69
	4.83		7.19	
	2.96		3.49	
8.44 E-1	1.23	1.54	3.62	3.36
	0.84		4.35	
	2.55		2.12	
NTC[b]	0		0	

a. Values reflect copies/μL of calculated serial dilution of positive plasmid DNA standard.

b. NTC means no template control.

Table S4 Quantitative results of qPCR and dPCR method in reproducibility comparison

Sample	qPCR value[a]		qPCR inter-assay CV%	dPCR value[b]		dPCR inter-assay CV%	CV% Decreased[c]
P6-1 (2.5E+3 copies/μL)	Test1	3098.68	24.21%	Test1	2583.38	0.63%	97.40%
	Test2	1992.09		Test2	2607.72		
	Test3	2199.08		Test3	2576.56		
P6-2 (1.25E+3 copies/μL)	Test1	1608.90	8.43%	Test1	1212.57	2.89%	65.72%
	Test2	1377.17		Test2	1144.91		
	Test3	1417.93		Test3	1173.2		
P7-1 (2.5E+2 copies/μL)	Test1	402.93	21.43%	Test1	234.91	0.98%	95.43%
	Test2	267.07		Test2	239.15		
	Test3	307.23		Test3	235.43		
P7-2 (1.25E+2 copies/μL)	Test1	231.55	30.60%	Test1	114.66	3.41%	88.86%
	Test2	124.91		Test2	113.17		
	Test3	168.94		Test3	120.65		
P8-1 (2.5E+1 copies/μL)	Test1	35.89	40.41%	Test1	20.62	5.11%	87.35%
	Test2	16.42		Test2	18.62		
	Test3	22.06		Test3	19.56		
P8-2 (1.25E+2 copies/μL)	Test1	25.89	27.12%	Test1	14.75	5.77%	78.72%
	Test2	14.93		Test2	15.81		
	Test3	24.01		Test3	14.11		
P9-1 (2.5E+0 copies/μL)	Test1	3.02	27.03%	Test1	2.02	18.95%	29.89%
	Test2	2.18		Test2	1.62		
	Test3	3.81		Test3	2.38		
P9-2 (1.25E+0 copies/μL)	Test1	1.80	53.08%	Test1	0.64	44.32%	16.50%
	Test2	3.77		Test2	1.33		
	Test3	1.46		Test3	0.67		

a Values reflect copies/μL based on serial dilutions of positive plasmid DNA standard.
b Values reflect copies/μL dPCR reaction.
c. Decreased CV% was calculated by 100%×(CV% qPCR-CV%d PCR)/ CV% qPCR.

Table S5 Quantitative results of qPCR and ddPCR method in inhibitor comparison

Inhibitor percentage[a]	qPCR assay				ddPCR assay	
	qPCR value[a]	Ct means	Copy number	Mean	Copy number	Mean
0	25.59	25.03	19748.40	29150.61	123.23	123.94
	24.72		34433.28		122.40	
	24.77		33270.15		126.18	
10%	25.10	25.11	27019.08	26881.72	113.44	119.25
	25.29		23953.88		120.01	
	24.95		29672.22		124.30	
20%	25.36	25.17	22880.21	25987.12	117.26	120.03
	25.16		25905.46		120.59	
	24.98		29175.69		122.24	
30%	25.23	25.17	24799.15	25859.97	121.97	124.96
	25.15		26148.23		122.55	
	25.12		26632.52		130.36	
40%	25.41	25.30	22164.65	23835.38	116.98	122.65
	25.30		23726.42		126.60	
	25.18		25615.07		124.36	
NTC[b]	N/A		0		0	

a. Proportion of inhibitor added in the reaction system.

b. NTC means no template control.

Table S6 Quantitative results of qPCR and ddPCR method in inhibitor comparison

Sample	Ct value by qPCR	Copy number by ddPCR (copies/μL)	Sample	Ct value by qPCR	Copy number (copies/μL)
1	35.68	2.66	21	34.80	1.43
2	34.73	3.95	22	34.71	2.07
3	35.01	2.05	23	35.31	3.42
4	34.88	2.53	24	35.43	2.59
5	34.99	3.19	25	34.92	1.86
6	34.10	3.16	26	34.91	1.96
7	35.79	2.24	27	34.09	1.21
8	34.81	2.85	28	34.85	2.06
9	34.37	6.7	29	35.36	1.05
10	34.06	4.85	30	34.36	1.3
11	34.67	3.31	31	34.06	3.28
12	34.35	3.29	32	34.52	3.51
13	34.10	2.49	33	35.33	0.32
14	34.13	2.87	34	36.01	0.9
15	34.85	2.12	35	35.28	1.39
16	33.86	2.05	36	35.27	0.63
17	34.73	1.39	37	35.51	0.96
18	34.10	0.9	NTC	N/A	0
19	34.59	2.54	NTC	N/A	0
20	33.93	0.91	NTC	N/A	0

Table S7 Quantitative results of qPCR and ddPCR method in different extraction method

Sample type	Serial number	qPCR assay		ddPCR assay	
		Ct value	Mean	Copy number	Mean
Pure cultured single PWN	1	30.61	30.73	16.17	15.05
		31.08		12.08	
		30.50		16.91	
	2	31.27	30.18	13.08	15.33
		31.43		13.59	
		27.83		19.32	
	3	31.47	29.79	12.93	13.16
		27.77		11.44	
		30.14		15.12	
	4	31.38	30.50	14.26	19.63
		30.68		22.57	
		29.44		22.07	
	5	33.04	33.34	11.05	8.76
		32.45		7.7	
		34.53		7.53	
Single PWN spicked in wood sample	6	35.86	34.78	1.84	1.96
		34.70		1.96	
		33.79		2.1	
	7	34.89	34.31	3	2.65
		34.08		1.95	
		33.95		3.01	
	8	35.88	31.32	0.91	1.23
		34.49		0.91	
		23.58		1.87	
	9	36.61	35.17	2.29	2.01
		33.97		2.02	
		34.95		1.73	
	10	37.56	35.91	0.96	0.92
		36.15		0.84	
		34.01		0.98	
NTC[a]		0		0	

a. NTC means no template control.

Table S8 Diagnostic results of qPCR and ddPCR in detection wood samples

Deceased pine trees			Symptomatic trees			Asymptomatic trees		
	Copy number of ddPCR	Ct values of qPCR		Copy number of ddPCR	Ct values of qPCR		Copy number of ddPCR	Ct values of qPCR
A01	470.87	23.99	B01	2.2	35.04	C01	0	0
A02	411	25.62	B02	8148.08	22.23	C02	0	0
A03	7025.74	20.66	B03	2.24	35.07	C03	0	0
A04	1.84	34.08	B04	1061.59	25.06	C04	0.87	0
A05	5269.05	20.48	B05	146.33	26.51	C05	0	0
A06	0	36.45	B06	1897.53	23.24	C06	2.06	35.61
A07	5876.52	20.91	B07	104.66	27.46	C07	0	
A08	587.53	22.60	B08	2170.6	22.85	C08	0	0
A09	643.64	23.80	B09	4554.81	21.60	C09	0	0
A10	2953.22	21.27	B10	2.14	34.69	C10	0	0
A11	11018.35	19.37	B11	4458.88	21.53	C11	1.19	36.81
A12	609.33	23.07	B12	1.86	34.93	C12	0	0
A13	5.21	30.68	B13	1745.77	23.21	C13	0.49	37.61
A14	0.64	34.95	B14	10646.18	19.42	C14	0.37	0
A15	76.04	25.86	B15	7739.11	20.58	C15	0	0
A16	875.63	23.64				C16	0	0
A17	1872.47	21.39				C17	0.19	0
A18	2538.3	21.83				C18	0	0
A19	735.19	23.11				C19	0.53	38.83
A20	783.43	23.61				C20	0	0
A21	5603.95	20.651				C21	0	0
A22	992.66	23.52				C22	0	0
A23	55.9	26.95				C23	0.23	37.32
A24	1383.84	23.27				C24	0	0
A25	1151.36	22.69				C25	0	0
A26	8216.14	20.41				C26	0	0
A27	0.97	34.66				C27	0	0
A28	765.63	23.66				C28	0.79	37.33
A29	3104.49	20.46				C29	0	0
A30	5760.48	21.27				C30	1.45	37.31
A31	147.86	26.61				C31	0	38.91
A32	8280.91	20.64				C32	18.98	30.90
A33	2775.93	21.66				C33	0	0
A34	19.09	29.06				C34	0	38.59
A35	3178.99	21.50				C35	0	0
A36	1107.56	22.39						

A37	1.19	34.88						

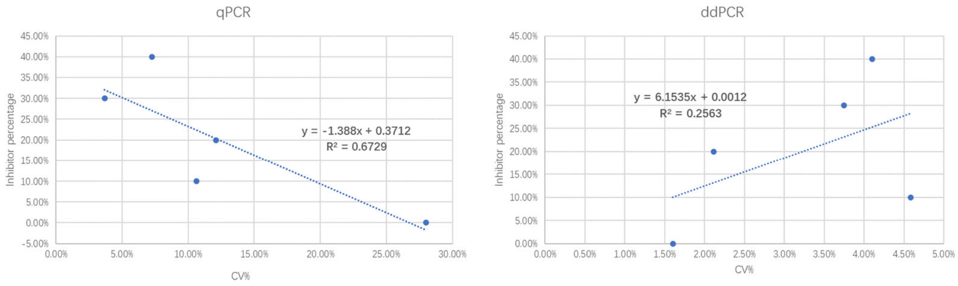

Figure S1 Linear regression of the CV% and inhibitor percentage for qPCR and ddPCR assays.
